# Annular pancreas as a cause of neonatal duodenal obstruction, a case report

**DOI:** 10.1093/jscr/rjad407

**Published:** 2023-07-21

**Authors:** Jorge Aurelio Gutiérrez-González, Emilia González De La Torre, Edgar Alan Armijo-Borjón, Abraham Alexander Alarcón-Sandoval, Francisco Javier Reyna-Sepulveda, Fernando Felix Montes-Tapia, Gerardo Enrique Muñoz-Maldonado

**Affiliations:** General Surgery Department, Hospital Universitario “Dr. José Eleuterio González” UANL, Monterrey, Mexico; Department of Pediatrics, Hospital Universitario “Dr. José Eleuterio González” UANL, Monterrey, Mexico; General Surgery Department, Hospital Universitario “Dr. José Eleuterio González” UANL, Monterrey, Mexico; General Surgery Department, Hospital Universitario “Dr. José Eleuterio González” UANL, Monterrey, Mexico; General Surgery Department, Hospital Universitario “Dr. José Eleuterio González” UANL, Monterrey, Mexico; General Surgery Department, Hospital Universitario “Dr. José Eleuterio González” UANL, Monterrey, Mexico; Department of Pediatrics, Hospital Universitario “Dr. José Eleuterio González” UANL, Monterrey, Mexico; General Surgery Department, Hospital Universitario “Dr. José Eleuterio González” UANL, Monterrey, Mexico

**Keywords:** annular pancreas, neonate, obstruction, case report

## Abstract

The annular pancreas (AP) is an uncommon congenital anomaly, characterised by a circumferential envelope in the second portion of the duodenum. In recent years, some genetic component has been found in the etiology. A newborn full-term male, weighing at 1910 g at birth, had a history of intrauterine growth restriction and diagnosis of tetralogy of Fallot, Down syndrome and congenital hypothyroidism. Duodenal membrane is suspected after persistent postprandial vomiting and abdominal distension; his abdomen was distended, hyperresonant and soft. The gastroduodenal series showed data compatible with a duodenal membrane so exploratory laparotomy was performed, finding the pancreas completely wrapping the second portion of the duodenum, so a diamond-shaped-duodenoduodenostomy anastomosis was performed. The AP should be considered, especially in male neonates with postprandial vomiting, abdominal distension, who show some other congenital anomaly, and in the abdominal X-ray, the sign of the double bubble is observed.

## INTRODUCTION

Annular pancreas (AP) is a rare congenital anomaly occurs in 1:20 000 live births. It is characterised by a circumferential envelope in the second portion of the duodenum, it is also described as a band of pancreatic tissue that conditions stenosis and obstruction, and its treatment is surgical [1, 2].

The AP was first recognised by Tiedman in 1818 and named as PA by Ecker in 1862 [3]. Its etiology is still unknown, but recently a genetic component has been found in the HNF1B and FOXF1 genes [4, 5].

## CASE REPORT

A newborn full-term male, with a weight of 1910 g and height of 43 cm, had a history of intrauterine growth restriction and diagnosis of tetralogy of Fallot, Down syndrome and congenital hypothyroidism. He was hospitalised at the pediatric intensive care unit for suspected duodenal membrane after persistent postprandial vomiting and abdominal distension.

Physical examination revealed a hypotonic newborn, normocephalic with phenotypic features of trisomy 21 and a systolic murmur. His abdomen was distended, hyperresonant and soft on palpation. The rest of the physical examination showed no abnormalities.

**Figure 1:**
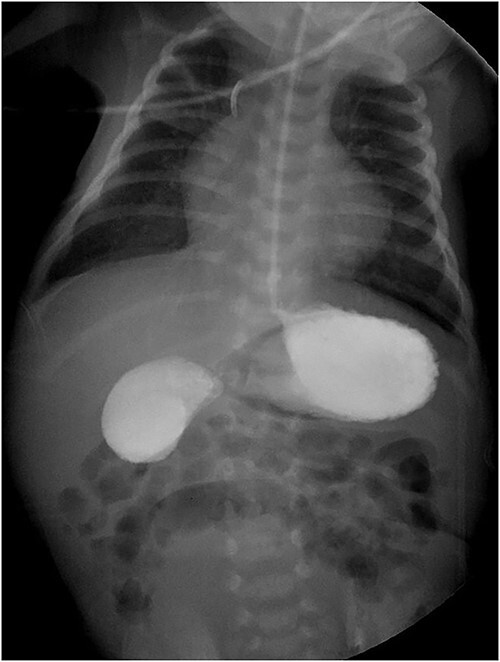
Gastroduodenal series showing the ‘double bubble’ sign.

**Figure 2:**
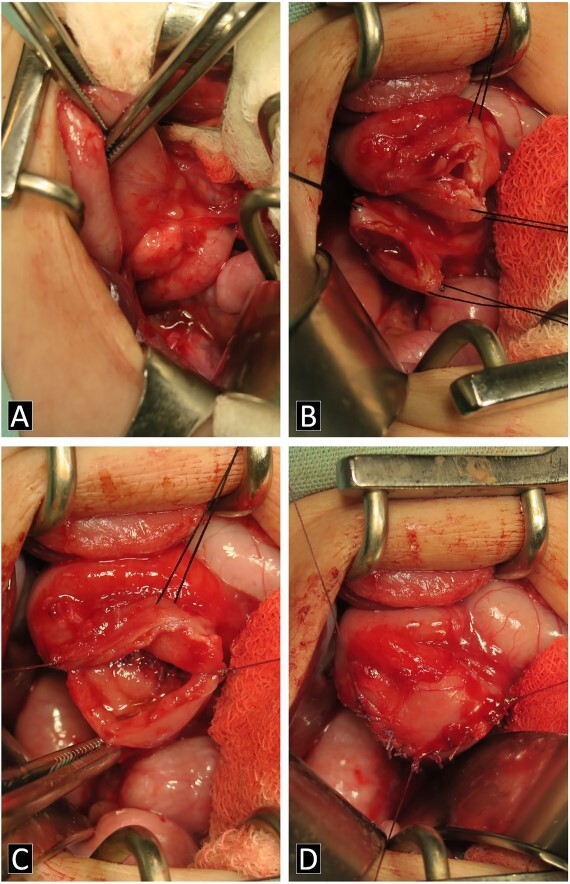
Intraoperatively images. (**A**) AP is shown in the second portion of the duodenum. (**B**–**D**) Process of the diamond-shaped-duodenoduodenostomy anastomosis.

The gastroduodenal series showed data compatible with a duodenal membrane between the second and third portions of the duodenum (Fig. 1). Based on the above, exploratory laparotomy was scheduled, where in the transoperative period, the pancreas was found completely wrapping the second portion of the duodenum (Fig. 2A), so it was decided to perform a diamond-shaped-duodenoduodenostomy anastomosis (Fig. 2B–D), which was performed without eventualities. He continued his postoperative period in neonatal intensive care, where after 5 days with nasogastric tube and fasting, the nasogastric tube was removed, and the oral diet was well tolerated. The patient was reviewed 1 week after discharge for the removal of surgical stitches; this was done at 3 and 6 months also, with adequate development and growth.

## DISCUSSION

Some series report that AP is more frequent in males, up to a 2:1 ratio [2, 3]; however, others describe it 1:1 [6]. Up to one-third of cases (16–28%) are born prematurely [2, 3, 6]. The average birth weight is usually 2566 g (1.9–3 kg), which could be due to the lack of absorption of amniotic fluid in the gastrointestinal tract in addition to impaired pancreatic insulin secretion [3]. In our case, we describe a male with a birth weight of 1.9 kg with a history of intrauterine growth restriction.

AP has been reported to be commonly associated with other congenital anomalies (27–67.5%), mainly cardiac anomalies, but also with Down syndrome, duodenal atresia, esophageal atresia, duodenal membrane, intestinal malrotation, Meckel’s diverticulum, anal atresia, among others [1–3, 6, 7].

Prenatal ultrasound usually shows abnormalities in up to 64% of cases, in particular the sign of fetal abdominal double bubble, polyhydramnios or some malformation of the digestive tract is described [2, 3, 6]. In the case described, we did not have the ultrasound results, but only the information about the intrauterine growth restriction that the mother verbally provided.

The clinical presentation varies according to the degree of obstruction, the main symptom is vomiting up to 59–94%, being mostly (2/3 parts) biliary and classically, postprandial. Abdominal distension, weight loss and, sometimes, hyperbilirubinemia are also described [2, 3, 6].

The diagnosis of duodenal obstruction is made with the clinical picture and the finding of the ‘double bubble’ sign on a plain abdominal X-ray, present in 90.9–100% of cases. It can be confirmed with a gastroduodenal series showing obstruction at the level of the duodenum. Some describe the use of computed tomography (CT) and magnetic resonance imaging (MRI). Despite the above, the gold standard is the complete examination of the duodenum and pancreatic head during surgery [2, 3, 8].

The treatment is surgical, the objective is to relieve the duodenal obstruction by means of a surgical bypass that restores the alimentary transit, either by gastrojejunal anastomosis, laterolateral duodenojejunal bypass or diamond-shaped duodeno-duodenal anastomosis. The latter is the most frequently used, as it is more physiological and is considered the best, in addition to presenting fewer complications [1–3].

Traditionally, this was performed by laparotomy, with a transverse supraumbilical incision; however, with the advent of laparoscopic surgery, it has become the gold standard, due to the shorter recovery and hospitalisation time, less pain, less risk of bleeding, and formation of adhesions and scarring. Its limitation is that it requires the skill of the surgeon and, even more, the availability of supplies [2, 9, 10].

In one study, a mean of 7.8+/−2.7 d of fasting after the surgical procedure was described. Regarding potential postsurgical complications, surgical site infection (2.6%) and anastomotic leakage (1%) were described [6]. In our case, there were 5 days of fasting and no complications were presented.
